# Overcoming Suppressive Tumor Microenvironment by Vaccines in Solid Tumor

**DOI:** 10.3390/vaccines11020394

**Published:** 2023-02-09

**Authors:** Ya-Jia Xie, Wen-Qian Liu, Dan Li, Jin-Cai Hou, Paolo Saul Coghi, Xing-Xing Fan

**Affiliations:** 1 State Key Laboratory of Quality Research in Chinese Medicine, Macau University of Science and Technology, Macau 999078, China; 2 Beijing Wante’er Biological Pharmaceutical Co., Ltd., No. 32 yard, East 2nd Road, Yanqi Economic Development Zone, Huairou District, Beijing 101400, China

**Keywords:** cancer vaccines, immunosuppressive TME, solid tumor, immunotherapy, nanovaccines

## Abstract

Conventional vaccines are widely used to boost human natural ability to defend against foreign invaders, such as bacteria and viruses. Recently, therapeutic cancer vaccines attracted the most attention for anti-cancer therapy. According to the main components, it can be divided into five types: cell, DNA, RNA, peptide, and virus-based vaccines. They mainly perform through two rationales: (1) it trains the host immune system to protect itself and effectively eradicate cancer cells; (2) these vaccines expose the immune system to molecules associated with cancer that enable the immune system to recognize and destroy cancer cells. In this review, we thoroughly summarized the potential strategies and technologies for developing cancer vaccines, which may provide critical achievements for overcoming the suppressive tumor microenvironment through vaccines in solid tumors.

## 1. Introduction

**Figure 1:**
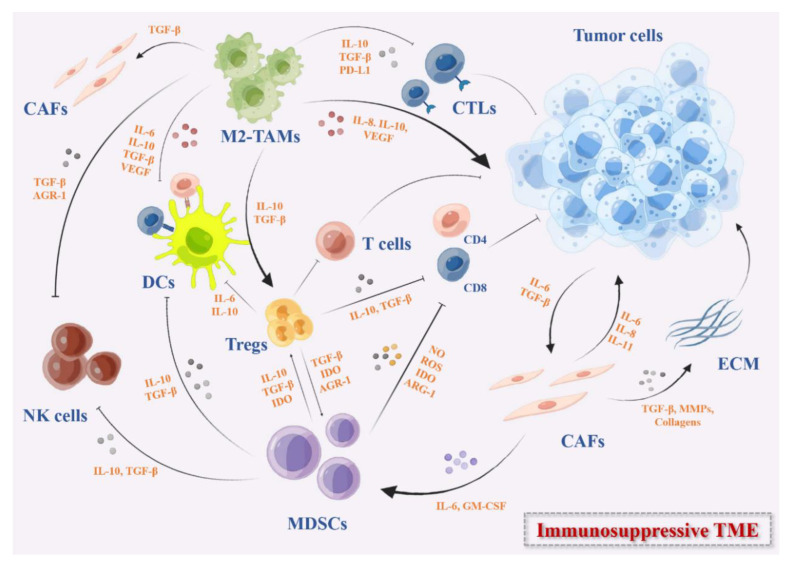
The immunosuppressive TME in solid tumors. These immunosuppressive cells include MDSCs, DCs, M2-TAMs, Tregs, and CAFs. They secrete immunosuppressive cytokines such as IL-10, IDO, TGF-β, growth factors such as VEGF, the checkpoints ligands such as PD-L1, or express checkpoints on the cell surface that can inhibit the activation of DC-mediated T cells and effector T cells directly or indirectly, remodel the ECM, and promote the angiogenesis in TME.

Vaccines provide a new opportunity for the prevention and treatment of infectious diseases. The pandemic of COVID-19 promoted the rapid development of vaccine technology and made cancer vaccines re-emerge in public focus [1]. Cancer vaccines are active immunotherapies that use nucleic acid sequences, peptides, proteins, and exosomes containing tumor-specific antigens (TSAs) or tumor-associated antigens (TAAs) to induce a specific immune response and eventually suppress tumor growth. With the successful identification of tumor antigens, personalized neoantigens vaccines and immune checkpoint inhibitors that reverse tumor-induced immune depletion, cancer vaccines have been regarded as a potentially promising therapeutic strategy in the immunotherapy of solid tumors [2]. However, the antitumor efficiency of cancer vaccines is weakened and impaired due to the highly immunosuppressive characteristics of the tumor microenvironment (TME) (Figure 1) [3,4]. In recent years, combined cancer vaccines with various immunotherapies or standardized therapies have become an effective strategy to reverse immunosuppressive TME and improve clinical outcomes [5,6]. Moreover, the availability and low cost of high-throughput sequencing technology have led to the identification of many tumor neoantigens. The in-depth research on immune mechanisms and various new vaccine platforms have widely promoted the research of cancer vaccines. In this review, we thoroughly discussed various potential tumor vaccines and its action mechanisms. Especially for solid tumors with immunosuppressive TME, we hope this review may help overcome this obstacle for cancer immunotherapy. 

### 1.1. Cell-Based Cancer Vaccines

Cell-based cancer vaccines are the main form of original cancer vaccine. For instance, dendritic cell [7] is a specialized antigen-presenting cell and plays a vital role in initiating a specific T cell response in innate antitumor immunity [8]. The dendritic cell-based [7] vaccine has achieved significant results in clinical trials. It is capable of presenting cancer antigens through MHC-I and MHC-II molecules, thereby initiating an antigen-specific immune response [9,10]. The first FDA-approved DC-based vaccine Sipuleucel-T was successfully used for the treatment of metastatic prostate cancer in 2020 [11]. Studies have indicated that Sipuleucel-T prolonged the overall survival of patients with prostate cancer and reduced the risk of death [12]. Although DCs inhibit tumor growth, tumor-infiltrating DCs usually show impaired or defective function in various tumors which exacerbate immunosuppressive effects and promote tumor development [13,14]. In addition, various types of immune cells such as tumor-associated macrophages (TAMs), myelogenous inhibitory cells (MDSCs), and regulatory T cells (Tregs) in TME also inhibit the effector T cell response and release cytokines to affect the function of DCs [15]. 

To enhance the anti-cancer immune response, many DC vaccines have been prepared and loaded with various TAAs or adjuvants to development of vaccines against TME, which mainly focuses on five categories: autologous dendritic cells, autologous dendritic cells loaded with tumor lysates, autologous DC transfected or pulsed with TAA-encoded RNA, autologous DC loaded with recombinant TAAs or TAA-derived peptides, and other DCs [16]. TAA targets are expressed at high levels in different tumor cells, and the most common TAAs include MUC1, WT1, CEA, mesothelin, and mutated KRAS [17]. It is generally suggested that immature DCs induce tolerance to itself, while mature DCs resist foreign antigens and exercise immune response. Therefore, stimulating mature DCs is the primary key factor for vaccine preparation [18]. The activation of DC vaccine currently mainly adopts “mature cocktail” therapy composed of proinflammatory cytokines TNF-α, IL-1β, IL-6, and Toll-like receptor agonists. The monocyte-derived DCs (MoDCs) exposure to a “maturation cocktail” while loaded with antigens enhances antigens capture, processing, and presentation on MHC I and MHC II molecules, increases the expression of co-stimulatory molecules CD80 and CD86, and induces DCs to initiate immature T cells [19].

The selection of appropriate antigens and antigens loading methods is crucial for DC vaccine to stimulate immune response. Common tumor antigens include tumor lysates, specific TAA-based peptides, protein, mRNA, and even whole tumor [20]. The whole tumor lysates contain a variety of tumor antigens, such as TSAs. However, other unrelated antigens are also present in the tumor lysates, resulting in decreased specificity that hinders antigens processing and presentation of DCs [21]. Peptide- or protein-based DC vaccines can reduce the incidence of autoimmune-related adverse reactions while maintaining tumor selectivity [17]. Peptides can be loaded directly onto MHC-I and MHC-II molecules on the DCs surface whereas protein and tumor cell MHC-I pathways are not specifically targeted and need to be processed and presented by DCs to induce T cells [22]. In contrast to peptide-based DC vaccines, the advantage of protein-based DC vaccines is not limited to selected haplotypes. Multiple epitopes appear on different haplotypes, thereby inducing an immune response against a broad spectrum of antigens [23]. Gene-edited DC is another effective antigen-loading method, transfecting mRNA encoding TSAs or TAAs into DCs, which avoids the need to identify haplotypes in patients and induces T cell immune response [10]. In addition, the combination of cancer vaccine with currently used cancer therapies such as radiotherapy, chemotherapy, immune checkpoint inhibitors (ICIs), and adoptive T cell therapy is an effective method to improve immunogenicity and inhibit the growth of malignant tumors. 

In conclusion, vaccines provide a very promising option for anti-cancer therapy. However, the existence of immunosuppressive TME makes it difficult for the DC vaccine to exert noteworthy antitumor immunity. To further improve the efficacy, we can innovate by optimizing the DCs maturation systems, selecting the appropriate antigens, optimizing the tumor antigens loading methods, and combining with other therapies (Table 1).

### 1.2. DNA-Based Vaccine 

DNA vaccines are now considered as a potential strategy to fight solid tumors by activating the immune system. Compared with traditional vaccines, DNA vaccines have shown great advantages in many aspects: (1) inducing both humoral immunity and cellular immunity; (2) simple and flexible design; (3) high safety, no pathogen infection risk, less adverse reactions; (4) and low cost and high production speed, and is suitable for large-scale production [24].

DNA vaccines are double-stranded nucleotides that encode a specific tumor antigen-encoding gene or immunostimulatory molecule that is transported to the host cell by a variety of delivery methods. DNA vaccines reach the cytoplasm through the cell membrane of APC and migrate to the nucleus for replication, transcription, and antigen production. The host cells express the target antigen and present the antigen through the MHC signaling pathway, thereby activating CD4^+^ T cells and CD8^+^ T cells and inducing immune responses [25]. DNA vaccines with built-in unmethylated CpG motif can bias the immune response to Th1, which is conducive to the induction of CTLs to kill the tumor, with a strong immune stimulation [26].

Although DNA vaccines have been shown to enhance antitumor immune responses, they are generally less immunogenic and less effective in clinical trials, primarily due to different resistance mechanisms during tumor development [27]. Therefore, optimizing the delivery system is essential to induce an effective immune response against tumor-associated antigens. The most common delivery methods of DNA vaccine are intradermal (ID) delivery and intramuscular (IM) delivery. Compared with IM delivery, ID delivery induces enhanced expression of antigens, leading to higher immunogenicity. Due to the high density of complex DCs network in dermis, the antigens are better exposed to DCs to initiate the immune response, thereby ID is the most suitable route for DNA delivery [28]. In recent years, several physical and chemical methods have been developed for DNA vaccine delivery, including gene gun delivery, electroporation, microneedles arrays, liposomes, virosomes, and nanoparticles [28,29]. Thus, optimizing the delivery system is a potential method to enhance the immunogenicity of DNA vaccines. 

In addition, adjuvants are used as immunostimulatory to enhance the immunogenicity of antigens, so the development of new DNA vaccine adjuvants also significantly affects the efficacy of DNA vaccines [30]. CpG oligonucleotide (CpG ODN) activates the innate immune system and increases the number of CD8^+^ T cells by binding to intracellular homologous TLR-9 receptors [31]. Many cytokines that enhance cellular and humoral immune responses have been used as DNA vaccine adjuvants such as chemokines, interleukins, granulocyte/macrophage colony-stimulating factor (GM-CSF), co-stimulatory molecules, and signaling molecules to induce the immune response via Th1 and Th2 cellular pathways [32]. Studies have revealed that codon-optimized GM-CSF linked to DNA vaccine boosts IFN-γ production in specific CD8^+^ T cells and CD4^+^ T cells and polarizes Th1 immune response [33]. The plenty of DNA vaccine experiments with adjuvants have been conducted in mice or other animals, but few experiments have been conducted in human bodies, thus pending further, more in-depth research and.

In general, DNA-based vaccines have become a useful tool for the treatment of cancer. The use of adjuvants and optimization of drug delivery systems have enabled DNA vaccines to better exert the immune mechanism. In addition, DNA vaccines combined with immunosuppressive agents or other immunotherapy has become a new trend in DNA vaccines in many clinical trials (Table 2).

### 1.3. RNA-Based Vaccine

The FDA approval of two kinds of COVID-19 mRNA vaccines (mRNA-1273 and BNT162b2) to respond to the COVID-19 pandemic has generated widespread interest in mRNA vaccines [34]. Similar to DNA vaccines, mRNA vaccines also induce both humoral and cellular immunity. Rationally, the mRNA encoding TSAs or TAAs enters the cytoplasm to bind with the ribosome of the host cell and translate. The antigenic proteins are degraded by the proteasome in the cytoplasm into antigenic peptides that are loaded onto MHC I for antigen-specific CD8 T cell activation. Cross-presentation of extracellular proteins on MHC I or loading onto MHC II activates CD4 T cells [35,36].

RNA vaccines have more advantages compared with DNA vaccine: mRNA is translated in splinter cells and non-splinter cells. Unlike DNA vaccines that need to migrate to the nucleus, mRNA only needs to be transferred into cytoplasm, and mRNA protein expression rate and quantity are generally higher than DNA vaccines; the mRNA vaccine is not integrated into the host genome sequence, and there is no risk of infection or insertion mutation [37,38]. However, there are some limitations in mRNA vaccines development. On the one side, the naked mRNA is rapidly degraded by extracellular RNases. On the other side, mRNA has inherent immunogenicity, which activates interferon-related reactions to further promote mRNA degradation, leading to decreased antigen expression [39].

The applications of mRNA vaccines have been limited by inefficient in vivo delivery. The mRNA are macromolecular substances that are unable to reach the cytoplasm through the lipid bilayer membrane of cell membrane, greatly limiting its clinical application. In order to solve the problem that mRNA is difficult to transmit through the cell membrane, different vectors have been developed to deliver mRNA, mainly including viral vectors, non-viral vectors, and dendritic cell-based vectors. Among many carriers, lipid nanoparticles (LNPs) are the most widely used delivery vehicles, which usually consist of four parts: (1) ionizable or cationic lipids for interaction with mRNA molecules; (2) auxiliary phospholipids similar to phospholipid bilayer; (3) cholesterol analog for stabilizing that LNP structure; (4) and polyethylene glycol (PEG) [40]. The ionizable lipid is a determining factor in the potency of the LNP, as it is positively charged at acidic pH and enhances the encapsulation of negatively charged mRNA by electrostatic interaction. In acidic environments, positively charged lipids interact with the ionic endosome membrane to promote membrane fusion and destabilization, resulting in mRNA release from the LNP and endosome [37]. However, ionizable lipids are essentially unchanged at physiological pH, which is a physiological property to promote endosome escape of mRNA.

A number of clinical studies have been conducted on mRNA packaged with LNP. The mRNA-4157 vaccine is a personalized mRNA vaccine encoding multiple antigens and delivering via LNP developed by Moderna in the United States [41]. Two clinical studies on the safety, tolerability, and immunogenicity of mRNA-4157 combined with pembrolizumab in the treatment of solid tumors are ongoing (NCT03313778/ NCT03897881). In this study, MRNA-4157 has shown remarkable safety and tolerability and induced potent antigen-specific T cell response.

Transfection of mRNA into DC was the first mRNA-based vaccine to enter clinical trials. At present, there are two delivery methods of DC-based mRNA vaccine, i.e., in vitro loaded DCs and in vivo targeted DCs. Although the procedure of ex vivo loading of DCs is complex and costly, it can achieve accurate antigen stimulation and high-efficiency transfection. DC-based mRNA vaccine is loaded in vitro by obtaining immature DCs from peripheral blood of patients, loading antigen-encoded mRNA after cells maturation, and returning to patients to initiate immune response and exert anti-cancer activity [42,43].

In a Phase I/II study, the immune response following vaccination with dendritic cells via mRNA electroporation with single-step antigen loading and TLR activation was explored in patients with stage III and IV melanoma. Participants were melanoma patients who demonstrated expression of melanoma-associated tumor antigen gp100 and tyrosinase. The results showed that intranodal administration of mRNA-optimized DC exerted great feasibility and safety, but limited TAA-specific immune response was observed (NCT01530698) [44]. In another Phase I/II trial of vaccine therapy with mRNA-transfected DCs in patients with advanced malignant melanoma, 16 of 31 patients showed tumor-specific immune responses, and the survival rate of those with responders was improved compared with non-responders. Most patients also respond to autologous DC antigens (NCT01278940) [45]. 

mRNA-based cancer vaccines have a broad prospect for cancer immunotherapy, but its potential has not been fully developed. With the development of nanotechnology, the use of vectors not only protects the mRNA from degradation, but also improves the immunogenicity of mRNA, making mRNA vaccine play a more effective anti-cancer mechanism. The adjustment of drug delivery routes and the combined delivery of multiple mRNA vaccines and other immunotherapeutic agents (such as checkpoint inhibitors) further improve the host antitumor immunity and increase the possibility of tumor cell eradication. Thereby, mRNA vaccine is a promising platform for cancer immunotherapy, which is expected to be rapidly developed for cancer immunotherapy in the near future (Table 3).

### 1.4. Peptide-Based Cancer Vaccines

Peptide-based cancer vaccines, usually consisting of a series of amino acids derived from tumor antigen or immune activating peptide from bacteria or other hosts, offer a strong immune stimulating effect [46,47]. The peptide-based vaccine has the advantages of convenient production, high speed, low carcinogenic potential, excellent safety profiles, insusceptible pathogen contamination, high chemical stability, low cost, and easy storage [46,48]. However, peptide-based vaccines are easily degraded by enzymes and have weak immunogenicity, which are difficult to induce robust and long-term immune response.

In order to promote the immunogenicity of peptide-based vaccines, it is important to optimize the sequence length of the peptide. Short peptides, approximately 8 to 12 amino acids in length, are presented without passing through a professional APC and directly bind to MHC I molecules of APCs, resulting in temporary T cell response and immune tolerance [49,50,51]. MHC II molecules can be combined with long peptides with a length of 12–20 amino acids. The peptides are assembled into peptide–MHC II complexes, which are delivered to the cell surface to be recognized by CD4^+^ T helper cells, triggering a specific T cell reaction and migrating to the tumor microenvironment to play an immune mechanism to inhibit tumor growth [50,52]. Therefore, long peptide vaccines are more likely to induce sustained and effective antitumor activity responses. 

The use of adjuvants protects the antigens from degradation and enhances specific immune response to antigens. TLR agonists have proven to be a promising adjuvant for peptide-based vaccines [53,54,55]. TLR is a pattern recognition receptor (PRR) that recognizes pathogen-associated molecular patterns (PAMPs). TLR is able to absorb antigens and provide key cytokines to stimulate and mediate TH1 and TH17 immune responses [53]. Studies have assembled new antigenic peptide and CpG ODN to form PCNPs nanocomposites, which are capable of simultaneously delivering new antigenic peptide and adjuvant to protect CpG ODN from nuclease-mediated degradation in serum, inducing effective an antigen presentation process and activating antigen-specific T cells [53,55]. Moreover, the combination of peptide vaccine with ICIs has achieved a very significant effect on tumor regression [7,56]. 

Although peptide-based cancer vaccines have specific cytotoxicity to tumor cells, there are significant challenges in inducing sustained and high level of immune response. We can hopefully overcome the immunosuppressive TME of peptide-based vaccines, effectively inhibit tumor immune evasion, and enhance antitumor activity by developing multi-target vaccines, optimizing adjuvants and nanomaterials, and combining with other therapies. Generally, peptide-based therapeutic cancer vaccine, is an alternative cancer immunotherapy, and possesses great potential for clinical application in the future (Table 4).

### 1.5. Virus-Based Cancer Vaccines 

Most viruses have natural immunogenicity, and their genetic material can be engineered to contain sequences encoding tumor antigens. Besides inducing local immune responses, local administration of many virus-based cancer vaccines also initiates systemic immune response, resulting in “abscopal effect”. The series of immune responses caused by virus infection eventually achieve effective and persistent antitumor immunity. Virus-based cancer vaccines are mainly divided into three forms: oncolytic virus vaccines, virus vector vaccines, and inactivated, live-attenuated or subunit vaccines against viruses that can induce tumors [57,58].

According to the report, an estimated 13% of cancers are related to viral infections in worldwide [59]. So far, hepatitis B virus (HBV), hepatitis C virus (HCV), human papillomavirus (HPV), merkel cell polyomavirus (MCV), Epstein–Barr virus (EBV), human herpesvirus type 8 (HHV-8), human T cell lymphotropic virus type 1 (HTLV-1), and human immunodeficiency virus (HIV) are common carcinogenic viruses in humans [60]. These DNA and RNA viruses produce carcinogenic effects via several different distinct mechanisms [61]. At present, many types of preventive vaccines have been used for HPV and HBV in clinical trials, but they provide limited benefits for eliminating pre-existing infections [62,63,64,65]. Moreover, therapeutic vaccines are urgently required to reduce the burden of the virus-related precancerous lesions and cancers.

Viruses are commonly used as vaccine vectors for gene delivery, owing to low cost and relative ease of production, purification, and storage [57]. The main types of virus vectors are adenovirus, alphavirus, poxviral (fowlpox, canarypox (ALVAC), vaccinia virus, and modified virus Ankara), and oncolytic virus (measles virus, herpes simplex virus (HSV), and vesicular stomatitis virus). Many studies have inserted TAAs, proinflammatory cytokines (GM-CSF, TNF-α, IL-2, IL-7, IL-12, and IL-23) and chemokines into the viral genome to intensify T cell activation and augment immune cell recruitment, leading to obtain better immune stimulation effects [66,67,68]. 

Oncolytic viruses, as an emerging immunotherapeutic agent, are able to expressly kill tumor cells and reverse immunosuppression by modulating TME components [69]. Talimogene laherparepvec (T-VEC), as a genetically modified herpes simplex oncolytic virus, was used in a phase II study of patients with unresectable stage IIIB-IV melanoma [70]. This study revealed that T-VEC induced systemic immune activity and revised the immunosuppressive TME, thus expanding the curative effect of other immunotherapeutic drugs in combination therapy [71,72]. 

Despite the immunomodulatory effect of virus-based cancer therapeutic agents, there are many limitations in immunotherapy. The approaches of antitumor immunity of virus-based vaccines require further investigation to achieve systemic delivery of therapeutic agents, potentiate efficacious immune responses, and minimize immune-mediated viral clearance. Collectively, multiple virus-based cancer vaccines have built a solid basis for treating malignancies in both preclinical and clinical studies (Table 5), a new era of anti-cancer therapy on virus-based cancer vaccines is expected in clinical trials.

### 1.6. Novel Bioactive Nanovaccines

The clinical outcomes of cancer vaccine have been largely hampered owing to the low antigen-specific T cell response rates and acquired drug resistance caused by the immunosuppressive TME. With the increasing understanding of the immunosuppressive mechanism of TME, it is feasible to combine nano technology with cancer vaccines and many associated clinical trials are undergoing (Table 6). 

The application of nanotechnology to tumor vaccines has effectively enhanced the efficacy of DC vaccines. The nano vaccine consists of antigens, adjuvants, and nano carriers. A variety of nanomaterials has been used to develop and design nanovaccines, including lipid-based NPs, protein-based NPs, natural NPs, polymer NPs, and others [88]. It has been reported that the PD-1 antibody based on nanotechnology solves the problems of difficult penetration of solid tumors and high cost and enhances the antitumor activity of tumor-specific CD8^+^ T cells [89].

Exosomes, as a novel biological nanocarrier, efficiently transfer proteins, lipids, and RNA between cells. Compared with nanomaterials, exosomes have the advantage that they can activate innate and adaptive immunity, and have better biocompatibility, biodegradability, and safety [89,90]. Tumor-associated exosome can effectively promote DC maturation and enhance MHC cross-presentation to reduce the expression of PD-L1 [91]. 

Some studies have indicated that the novel treatment regimens and combined immunotherapy used in the bioactive nanovaccine platform provides a new and effective treatment strategy in the therapy of solid tumors. Recently, a pH-sensitive antitumor nanovaccine has been reported, which encapsulated colony stimulating factor 1 receptor (CSF1-R) inhibitor BLZ-945 and indoleamine 2,3-dioxygenaseinhibitor NLG-919 in its core and displayed a model antigen ovalbumin on its surface [7]. This nanovaccine was used to remodel the immunosuppressive TME and thus expand DCs recruitment, differentiation, antigen presentation, and T cells response [79]. TME enriches with plentiful extracellular matrix (ECM) is a compact physical barrier for the penetration of immune cells. Hyaluronan (HA) is a critical component of the ECM, which is overexpressed in various tumors and is highly related to tumor proliferation, invasion, metastasis, migration, and radiochemotherapy resistance. Studies have combined tumor nanovaccine with hyaluronidase HAase gene therapy to activate BMDCs, enhance the specific reaction of T cells in vivo, and degrade tumor ECM, thus promoting the infiltration of immune cells and modulating the immunosuppressive microenvironment [81]. Cancer-associated fibroblasts (CAFs), the major cells of depositing and remodeling ECM in solid tumors, have been widely described as critical actors in tumor growth, metastasis, immunosuppression, and drug resistance. Fibroblast activation protein-α (FAP) is a transmembrane serine protease and is highly expressed on CAFs in most types of tumor tissues. FAP-positive CAFs (FAPCAFs) can recruit Tregs and promote their differentiation and proliferation into Tregs in various CAFs, producing an immunosuppressive TME [92,93,94]. Some researchers prepared an FAP gene-engineered tumor cell-derived exosome-like nanovesicles (eNVs-FAP) vaccine, which not only suppressed tumor growth by enhancing the infiltration of effector T cells in tumor cells and FAPCAFs and reprogramming the immunosuppressive TME, but also facilitated IFN-γ-induced tumor cell ferroptosis [82]. 

At present, although tumor nanovaccines have potential applications in the prevention and treatment of solid tumors, the therapeutic effects are generally limited due to the multiple immunosuppressive TME. Thus, the combination of nanovaccines and ICIs therapies is a potential effective strategy to induce antitumor immune response in vivo and relieve tumor immune tolerance microenvironment. A multifunctional biomimetic nanovaccine based on photothermal and weak-immunostimulatory nanoparticulate cores CCM@ (PSiNPs@Au) has been reported to activate DCs and the downstream antitumor immunity. In addition, combined with ICIs immunotherapy, this nanovaccine significantly suppressed the growth and metastasis of established solid tumors through initiating antitumor immune responses and reversing immunosuppressive TME to an immunoresponsive one [83]. Studies have indicated that the combination of mannosylated nanovaccines and gene-regulated PD-L1 blockade is able to target DCs and enhance antitumor immune response, thereby improving the efficacy of tumor vaccines and inhibiting tumor growth [84]. It has been exhibited that immunogenic cell death (ICD) is capable of activating the immune microenvironment to enhance the ICIs immunotherapy efficacy [95]. Recently, a self-amplified biomimetic nanosystem, mEHGZ, was prepared by was prepared by encapsulating epirubicin (EPI), glucose oxidase (Gox), and hemin in zeolitic imidazolate framework (ZIF-8) nanoparticles and coating with calreticulin (CRT) over-expressed tumor cell membrane. This mEHGZ nanovaccine amplified the ICD effect to promote DCs maturation and CTLs infiltration, thus intensifying the sensitivity of tumor cells to the treatment with anti-PD-L1 antibody [85]. 

Overall, these biomimetic nanoplatforms provide a novel promising method for improving the response rate of ICIs and reversing immunosuppressive TME. 

## 2. Conclusions

**Figure 2:**
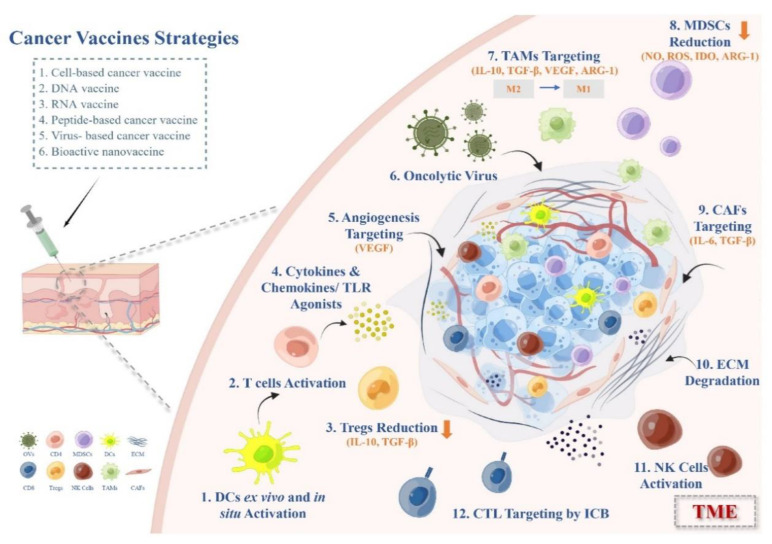
Therapeutic strategies of cancer vaccines overcoming immunosuppressive TME in solid tumors.

Therapeutic cancer vaccines have undergone a resurgence in the past decade. In this review, we thoroughly summarized the strategies and ideas for the exploitation of efficient cancer vaccine immunotherapy and discussed the action mechanisms and optimization of the clinical usage of distinct cancer vaccines for the treatment of solid tumors in the immunosuppressive microenvironment (Figure 2). Various types of vaccine platforms and adjuvants provide feasibility for tumor vaccine development.

Cancer therapeutic vaccines are capable of initiating cancer-specific immune responses with minimal adverse autoimmunity, which not only induce localized immune responses, but also remodel the immunosuppressive TME, leading to the synergy with other immunotherapy methods. The aim of therapeutic cancer vaccines is to direct the immune system to induce tumor regression, eradicate minimal residual disease, establish persistent antitumor memory, and avoid non-specific or adverse reactions. However, due to the immunosuppressive properties of the TME in solid tumor, the antitumor potential of these vaccines is attenuated, posing major challenges to achieve this goal. 

Finally, we discussed recent emerging bioactive nanovaccines and their therapeutic strategies in immunosuppressive TME. Nanoparticles have provided distinctive opportunities to improve the immunotherapy effect of cancer vaccines. Nanovaccines remarkably expand the immunogenicity of vaccines and boost antigen-specific adaptive immune responses for cancer therapy via effectively co-delivering multivalent molecular antigens and adjuvants to lymphoid tissues and immune cells. Bioactive nanovaccines are prospective to maximize the potential of cancer vaccines in solid tumor and provide a very promising strategy for elevating the response rate of ICIs and reversing immunosuppressive TME. 

## Figures and Tables

**Table 1:** DC-based cancer vaccines in clinical application.

Category	Intervention	Conditions	Status	Phases	Trial No.
DC	PEP-DC Vaccine	Non-small Cell Lung Cancer	Recruiting	I	NCT05195619
DC	DC Vaccine Subcutaneous Administration	Gastric cancer, Hepatocellular Carcinoma, Non-Small-Cell Lung Cancer	Recruiting	I	NCT04147078
DC	KRAS-EphA-2-CAR-DC	Solid Tumor	Recruiting	I	NCT05631899
DC	mDC3/8-KRAS Vaccine	Pancreatic Ductal Adenocarcinoma	Recruiting	I	NCT03592888
DC	Autologous DC Vaccine	Pancreatic Adenocarcinoma	Recruiting	I	NCT04157127
DC	TP53-EphA-2-CAR-DC	Solid Tumor	Recruiting	I	NCT05631886
DC	PEP-DC Vaccine	Pancreatic Adenocarcinoma	Recruiting	I	NCT04627246
DC	Autologous Dendritic Cell-Adenovirus CCL21 Vaccine Pembrolizumab	Lung Non-Small Cell Carcinoma	Recruiting	I	NCT03546361
DC	Dendritic Cell Tumor Cell Lysate Vaccine Pembrolizumab	Recurrent Glioblastoma	Recruiting	I	NCT04201873
DC	Dendritic Cell (DC1) Vaccine	HER2-Positive Breast Cancer	Recruiting	I	NCT05378464
DC	HER2—Primed Dendritic Cells HER3—Primed Dendritic Cells	Triple-Negative Breast Cancer HER2-Negative Breast Cancer	Recruiting	I	NCT05504707
DC	Autologous Dendritic Cells Pulsed With Multiple Neoantigen Peptides	Glioblastoma Multiforme of Brain	Recruiting	I	NCT04968366
DC	MesoPher Mitazalimab	Metastatic Pancreatic Cancer	Recruiting	I	NCT05650918
DC	TTRNA-DC Vaccines with GM-CSF TTRNA-xALT	Diffuse Intrinsic Pontine Glioma (DIPG) Brain Stem Glioma	Recruiting	I	NCT03396575
DC	Dendritic Cell Vaccination + Temozolomide-Based Chemoradiation Dendritic cell Vaccination +- Conventional Next-Line Treatment	High Grade Glioma	Recruiting	I/II	NCT04911621
DC	Depletion of Treg+ DC Vaccine	Childhood Glioblastoma	Recruiting	I/II	NCT03879512
DC	ADC Vaccine	Extensive-Stage Small Cell Lung Cancer	Recruiting	I/II	NCT04487756
DC	Dendritic Cells Vaccine	Glioblastoma	Recruiting	I/II	NCT04801147
DC	Dendritic Cell/Tumor	Glioblastoma	Recruiting	I/II	NCT04388033
DC	Neoantigen-Expanded Autologous DC-CIK Cells	Advanced Solid Tumor	Recruiting	I	NCT05020119
DC	Autologous Dendritic Cells	Mesothelioma, Malignant	Recruiting	I	NCT03546426
DC	Autologous DC Vaccine	Head Neck Tumors Neuroendocrine Tumors	Recruiting	II	NCT04166006
DC	Neoantigen Dendritic Cell Vaccine	Hepatocellular Carcinoma	Recruiting	II	NCT04912765
DC	TCR-T Therapy	Pancreatic Cancer	Recruiting	Early I	NCT05438667
DC	Anti-HER2/HER3 Dendritic Cell Vaccine Pembrolizumab	Breast Cancer	Recruiting	II	NCT04348747
DC	HER-2 pulsed DC1	HER2-Positive Breast Cancer	Recruiting	II	NCT05325632
DC	Dendritic Cell Vaccine (DC1)	Breast Cancer	Recruiting	Early I	NCT03387553
DC	Camrelizumab plus GSC-DCV Camrelizumab Plus Placebo	Recurrent Glioblastoma	Recruiting	II	NCT04888611
DC	Chimeric Exosomal Tumor Vaccines	Recurrent or Metastatic Bladder Cancer	Recruiting	Early I	NCT05559177
DC	Pneumococcal 13-valent Conjugate Vaccine Therapeutic Autologous Dendritic Cells	Hepatocellular Carcinoma	Recruiting	Early I	NCT03942328

**Table 2:** DNA-based cancer vaccines in clinical application.

Category	Biological	Conditions	Status	Phases	Trial No.
DNA	PROSTVAC V/F	Metastatic Hormone-Sensitive Prostate Cancer	Completed	I	NCT03532217
DNA	pTVG-HP, pTVG-AR	Castration-resistant Prostate Cancer	Recruiting	II	NCT04090528
DNA	pTVG-HP	Prostate Cancer	Active, not recruiting	II	NCT03600350
DNA	pTVG-AR	Prostate Cancer	Recruiting	I/II	NCT04989946
DNA	GNOS-PV01	Glioblastoma	Active, not recruiting	I	NCT04015700
DNA	VXM01	Recurrent Glioblastoma	Active, not recruiting	I/II	NCT03750071
DNA	CD105/Yb-1/SOX2/CDH3/MDM2-polyepitope Plasmid DNA vaccine	Breast Cancer, Lung Non-Squamous Non-Small Cell Carcinoma	Recruiting	II	NCT05455658NCT05242965
DNA	pUMVC3-IGFBP2-HER2-IGF1R Plasmid DNA Vaccine	Breast Cancer	Recruiting	II	NCT04329065
DNA	MV-s-NAP	Breast Cancer	Recruiting	I	NCT04521764
DNA	pING-hHER3FL	Advanced Cancer	Recruiting	I	NCT03832855
DNA	SCIB1	Malignant Melanoma	Recruiting	II	NCT04079166
DNA	IFx-Hu2.0	Cutaneous Melanoma	Completed	Early I	NCT03655756
DNA	MEDI4736	Extensive-Stage Small Cell Lung Cancer	Recruiting	II	NCT04397003
DNA	GNOS-PV02 and INO-9012	HCC	Recruiting	I/II	NCT04251117
DNA	GRT-C901/GRT-R902	Colorectal Neoplasms	Recruiting	II/III	NCT05141721
DNA	MEDI0457	Carcinoma	Active, not recruiting	II	NCT03439085

**Table 3:** RNA-based cancer vaccines in clinical application.

Category	Biological	Conditions	Status	Phases	Trial No.
mRNA	W_ova1	Ovarian Cancer	Active, not recruiting	I	NCT04163094
mRNA	PGV002	Gastric Cancer	Recruiting	Not Applicable	NCT05192460
mRNA	BNT113	Carcinoma, Squamous Cell, Head and Neck Neoplasm	Recruiting	I/II	NCT03418480
mRNA	RNA tumor vaccine, RNA tumor vaccine+Navuliumab	Advanced Solid Tumor	Recruiting	I	NCT05202561
mRNA	mRNA-1273	Solid Tumor Malignancy	Recruiting	II	NCT04847050
mRNA	BNT113 Pembrolizumab	Unresectable Head and Neck Squamous Cell Carcinoma	Recruiting	II	NCT04534205
mRNA	SW1115C3	Solid Tumor	Recruiting	I	NCT05198752
mRNA	mRNA-4157 Pembrolizumab	Melanoma	Active, not recruiting	II	NCT03897881
mRNA	BNT111 Cemiplimab	Melanoma	Recruiting	II	NCT04526899
mRNA	RNA-LPs	Adult Glioblastoma	Recruiting	I	NCT04573140

**Table 4:** Peptide-based cancer vaccines in clinical application.

Category	Biological	Conditions	Status	Phases	Trial No.
Peptide	KRAS Peptide Vaccine+ Poly-ICLC	High Risk Cancer, Pancreatic Cancer	Recruiting	I	NCT05013216
Peptide	KRAS Peptide Vaccine+ Poly-ICLC	Colorectal Cancer, Pancreatic Cancer	Recruiting	I	NCT04117087
Peptide	ESR1 Peptide Vaccine	Breast Cancer	Recruiting	I	NCT04270149
Peptide	Pooled Mutant KRAS-Targeted Long Peptide Vaccine	Non-Small Cell Lung Cancer	Recruiting	I	NCT05254184
Peptide	Neoantigen Peptides	Neoplasms	Recruiting	Early I	NCT05475106
Peptide	Incomplete Freund’s Adjuvant Sargramostim SVN53-67/M57-KLH Peptide Vaccine	Lung Atypical Carcinoid Tumor, Lung Typical Carcinoid Tumor, Metastatic Pancreatic, Neuroendocrine Tumor	Recruiting	I	NCT03879694
Peptide	PGV-001 Poly-ICLC CDX-301	Prostate Cancer	Recruiting	I	NCT05010200
Peptide	OTSGC-A24	Gastric Cancer	Recruiting	I	NCT03784040
Peptide	Optimized Neoantigen synthetic Long Peptide vaccine+ Poly-ICLC	Pancreas Cancer	Recruiting	I	NCT05111353
Peptide	Neoantigen Peptide Vaccine Nivolumab	Breast Cancer	Recruiting	I	NCT05098210
Peptide	PolyPEPI1018	Metastatic Colon Adenocarcinoma	Recruiting	I	NCT05130060
Peptide	Autologous Heat Shock Protein 70 and Autologous Activated Monocytes	Hepatocellular Carcinoma	Recruiting	I	NCT05059821
Peptide	Neoantigen Peptide Vaccine Pembrolizumab Sargramostim	Breast Cancer	Recruiting	I	NCT05269381
Peptide	DNAJB1-PRKACA Peptide Vaccine	Fibrolamellar Hepatocellular Carcinoma (FLC)	Recruiting	I	NCT04248569
Peptide	H3K27M Peptide Vaccine	Newly Diagnosed H3-mutated Glioma	Recruiting	I	NCT04808245
Peptide	IDH1R132H Peptide Vaccine	Malignant Glioma	Recruiting	I	NCT03893903
Peptide	iNeo-Vac-P01	Resectable Pancreatic Cancer	Recruiting	I	NCT04810910
Peptide	GM-CSF+ H2NVAC	Breast Ductal Carcinoma In Situ	Recruiting	I	NCT04144023
Peptide	6MHP+ NeoAg-mBRAF	Melanoma	Recruiting	I/II	NCT04364230
Peptide	Personalized Neoantigen Vaccine	Pancreatic Tumor	Recruiting	I	NCT03558945
Peptide	iNeo-Vac-P01	Advanced Malignant Solid Tumor	Recruiting	I	NCT04864379
Peptide	6MHP	Melanoma	Recruiting	I/II	NCT03617328
Peptide	Multipeptide Vaccine+ XS15	Chronic Lymphocytic Leukemia	Recruiting	I	NCT04688385
Peptide	Durvalumab Personalized Synthetic Long Peptide Vaccine Tremelimumab	Breast Cancer, Invasive Breast Carcinoma, Metastatic Triple-Negative Breast Carcinoma	Recruiting	II	NCT03606967
Peptide	EO2040	Colorectal Cancer	Recruiting	II	NCT05350501
Peptide	Multi-epitope HER2 Peptide Vaccine TPIV100 Pertuzumab	Breast Adenocarcinoma	Recruiting	II	NCT04197687
Peptide	PolyPEPI1018	Colorectal Cancer Metastatic	Recruiting	II	NCT05243862
Peptide	UCPVax	Squamous Cell Carcinoma of the Head and Neck	Recruiting	II	NCT03946358
Peptide	UCPVax	Glioblastoma	Recruiting	II	NCT04280848
Peptide	SurVaxM	Newly Diagnosed Glioblastoma	Recruiting	II	NCT05163080
Peptide	IO102 IO103	Oropharynx Squamous Cell Carcinoma	Recruiting	II	NCT04445064
Peptide	Neoantigen Peptide	Pancreas Cancer	Active, not recruiting	I	NCT03956056
Peptide	AE37 Peptide Vaccine Pembrolizumab	Triple-negative Breast Cancer	Active, not recruiting	II	NCT04024800
Peptide	Neoantigen Peptides	Neoplasms	Completed	Early I	NCT04509167
Peptide	iNeo-Vac-P01	Pancreatic Cancer	Completed	I	NCT03645148
Peptide	Galinpepimut-S	Acute Myelogenous Leukemia, Ovarian Cancer, Colorectal Cancer	Active, not recruiting	I/II	NCT03761914
Peptide	iNeo-Vac-P01	Advanced Malignant Solid Tumor	Active, not recruiting	I	NCT03662815
Peptide	S-488210 S-488211	Lung Cancer, Head and Neck Cancer, Bladder Cancer	Completed	I	NCT04316689
Peptide	Peptide pulsed Dendritic cell	Breast Cancer Female	Completed	I	NCT04879888
Peptide	Bcl-Xl_42-CAF09b Vaccine	Prostate Cancer	Completed	I	NCT03412786
Peptide	EVAX-01-CAF09b	Malignant Melanoma, Non-Small Cell Lung Cancer	Active, not recruiting	I/II	NCT03715985
Peptide	PolyPEPI1018 CRC Vaccine	Colorectal Cancer	Completed	I/II	NCT03391232
Peptide	GEN-009 Adjuvanted Vaccine	Cutaneous Melanoma, Non-small Cell Lung Cancer	Completed	I/II	NCT03633110

**Table 5:** Virus-based cancer vaccines and their efficacy on the solid TME.

Category	Product name	Conditions	Strategy	Efficacy	Trial No.	Reference
Oncolytic viruses	T-VEC	Melanoma	Genetic engineering vector uses attenuated HSV coding to generate GM-CSF.	Induce systemic immune activity to revise the immunosuppressive TME.	NCT00769704	[71,72,73]
Oncolytic viruses	VV_GM_-αhCTLA-4 (BT-001)	Pan-cancer	Genes encoding the 4-E03 human recombinant anti-hCTLA4 antibody and human GM-CSF.	Induce Treg depletion and CD8^+^ T cell immunity	NCT04725331	[74]
Oncolytic viruses	YST-OVH	Hepatoma	Genes encoding a humanized scFv against human PD-1.	Augment the effector and memory CD8^+^ T cells and reduce the recruitment of MDSCs, and overcome localized immunosuppression to sensitize tumors to CTLA-4 or TIM-3 blockade.	No	[75]
Virus vector	Vvax001	Malignant Cervical Lesions	Combination of sunitinib, local tumor irradiation and therapeutic immunization.	Decrease intratumoral MDSCs and increase CD8^+^ and E7-specific T cell levels and activity.	NCT03141463	[76]
Virus vector	PRGN-2009	HPV-Positive Cancer	Containing multiple cytotoxic T cell epitopes of the viral oncoproteins HPV 16/18 E6 and E7.	Generate high levels of HPV16 E6-specific T cells and augment multifunctional CD8^+^ and CD4^+^ T cells in the TME.	NCT04432597	[77]
Virus vector	VRP-HER2	Breast Cancer	Alphaviral vector encoding HER2.	Induce HER2-specific memory CD8^+^ T cells and antibodies to inhibit tumor growth.	NCT03632941	[78]

**Table 6:** The novel strategies of bioactive nanovaccines in immunosuppressive TME.

Nanovaccine	Strategy and Method	Efficacy	Reference
BN@HM-OVA	Encapsulate inhibitor BLZ-945 and NLG-919 using hybrid micelles	Remodel the immunosuppressive TME via causing M2-like TAMs depletion and suppressing IDO activity	[79]
BCNCCM	Co-encapsulation of BP-Au-CpG and NLG919 by CCM	Induce immunogenic cell death and suppress the activities of Tregs to enhance immunotherapy efficacy	[80]
PEI/CaCO/OVA/CpG NVs and pSpam1@NPs	Nanovaccines combine with gene-mediated ECM scavenger	Degrade the tumor ECM and promote the infiltration of immune cells	[81]
eNVs-FAP	FAP gene-engineered tumor cell-derived exosome-like vesicle vaccines	Increase the infiltration of effector T cells and promote interferon-gamma-induced tumor cell ferroptosis	[82]
CCM@(PSiNPs@Au)	Combine biomimetic nanovaccines based on photothermal and weak-immunostimulatory nanoparticulate cores with ICB immunotherapy	Activate DCs and antitumor immune responses to reverse immunosuppressive TME	[83]
Man-PLL-RT/OVA/CpG and HA/PLL-RT/shPD-L1 NPs	Combine mannose receptor-mediated nanovaccines and gene-regulated PD-L1 blockade	Promote the endocytosis, maturation and cross presentation in DCs and relieve tumor immune tolerance microenvironment	[84]
mEHGZ	CRT over-expressed tumor cell membranes coating ZIF-8 nanoparticles loaded EPI, Gox and hemin	Induce cascade-amplified ICD effect and improve the sensitivity of aPD-L1 therapy	[85]
MPDA-R848@CM	Based on the surgical tumor-derived CMs coating R848 loaded MPDA photothermal nanovaccines	Combine with aPD-L1 therapy to enhance DCs activation and maturation, and stimulate antigen-specific CD8^+^ T cells.	[86]
DBE@CCNPs	The CD47KO/CRT dual-bioengineered cell membrane-coated PEI25k/CpG-NPs	Enhance the immunogenicity of tumor antigens and activate DCs to stimulate tumor-specific effector CD8^+^ T cells	[87]

## Data Availability

No new data were created or analysed. Data sharing is not applicable to this article.

## Abbreviations

TSAs	tumor-specific antigens
TAAs	tumor-associated antigens
TME	tumor microenvironment
DC	dendritic cell
TAMs	tumor-associated macrophages
MDSCs	myelogenous inhibitory cells
Tregs	regulatory T cells
MoDCs	monocyte-derived DCs
ICIs	immune checkpoint inhibitors
ID	intradermal
IM	intramuscular
CpG ODN	CpG oligonucleotide
GM-CSF	granulocyte/macrophage colony-stimulating factor
LNP	lipid nanoparticles
PEG	polyethylene glycol
PRR	pattern recognition receptor
PAMP	pathogen-associated molecular patterns
HBV	hepatitis B virus
HCV	hepatitis C virus
HPV	human papillomavirus
MCV	merkel cell polyomavirus
EBV	Epstein–Barr virus
HHV-8	human herpesvirus type 8
HTLV-1	human T cell lymphotropic virus type 1
HIV	human immunodeficiency virus
HSV	herpes simplex virus
T-VEC	talimogene laherparepvec
CSF1-R	colony stimulating factor 1 receptor
ECM	extracellular matrix
HA	hyaluronan
CAFs	cancer-associated fibroblasts
FAP	fibroblast activation protein-α
FAPCAFs	FAP-positive CAFs
eNVs-FAP	FAP gene-engineered tumor cell-derived exosome-like nanovesicles
ICD	immunogenic cell death
EPI	encapsulating epirubicin
Gox	glucose oxidase
ZIF-8	hemin in zeolitic imidazolate framework
CRT	calreticulin
